# Effect of silver on the phase transition and wettability of titanium oxide films

**DOI:** 10.1038/srep32171

**Published:** 2016-08-30

**Authors:** Adolfo A. Mosquera, Jose M. Albella, Violeta Navarro, Debabrata Bhattacharyya, Jose L. Endrino

**Affiliations:** 1 Instituto de Ciencia de Materiales de Madrid, Consejo Superior de Investigaciones Científicas, E-28049 Madrid, Spain; 2 Interface Physics Group, Kamerlingh Onnes Laboratory, Leiden University, The Netherlands; 3 Department, School of Aerospace, Transport and Manufacturing (SATM), Cranfield University, College Road, Cranfield, Bedfordshire MK43 0AL, UK

## Abstract

The effect of silver on the phase transition and microstructure of titanium oxide films grown by pulsed cathodic arc had been investigated by XRD, SEM and Raman spectroscopy. Following successive thermal annealing up to 1000 °C, microstructural analysis of annealed Ag-TiO_2_ films reveals that the incorporation of Ag nanoparticles strongly affects the transition temperature from the initial metastable amorphous phase to anatase and stable rutile phase. An increase of silver content into TiO_2_ matrix inhibits the amorphous to anatase phase transition, raising its temperature boundary and, simultaneously reduces the transition temperature to promote rutile structure at lower value of 600 °C. The results are interpreted in terms of the steric effects produced by agglomeration of Ag atoms into larger clusters following annealing which hinders diffusion of Ti and O ions for anatase formation and constrains the volume available for the anatase lattice, thus disrupting its structure to form rutile phase. The effect of silver on the optical and wetting properties of TiO_2_ was evaluated to demonstrate its improved photocatalytic performance.

Titanium dioxide is a polymorphous compound with a broad range of applications in catalysis and photocatalysis, gas sensors, energy storage, self-cleaning devices, optical and corrosion protective coatings^1^. These applications are dependent on the crystallographic structure, morphology and physical properties of the different phases of titania. As a bulk material, it can exhibit anatase, rutile and brookite forms while TiO_2_ thin films only show amorphous, anatase or rutile structures. Brookite thin films can only be achieved by chemical deposition techniques^2^,^3^ at high deposition or annealing temperatures^4^.

Different chemical routes have been used to synthesize bulk titania. Sol-gel^5^, hydrothermal techniques^6^, spray pyrolysis and chemical vapour deposition allow to synthesize anatase powders at low temperatures^7^,^8^, while rutile phase can be obtained after annealing treatments at temperatures around 900 °C^9^. In TiO_2_ powders, the transition temperature is determined by grain size, deposition techniques and process conditions such as thermal annealing^10^. In some applications, for instance optical coatings, the fabrication of rutile at lower temperatures is of paramount importance, since rutile is thermodynamically the most stable phase of titania, giving the highest refractive index and hardness^11^,^12^. Nowadays, there are many reports on the structural and morphological properties of the anatase-rutile (A-R) transformation upon annealing treatments, showing either pure or mixed rutile phase at temperatures above 800 °C^13^,^14^,^15^.

To promote the A-R transition at lower temperatures for bulk titania, many dopants have been proposed, thus expanding its range of applications. For instance, V-doped TiO_2_ powders show the promotion of rutile phase at 700 °C, with an improvement of their ferromagnetic characteristics^16^. Besides, Ni- and Ru-doped TiO_2_ systems also show rutile phase at 700 °C having larger grain size^17^. In addition, other dopant promoters such as Co, Cr, Cu, Na, Ni, Sn, Al and Zn have been studied in the past^12^. In these earlier studies, silver (Ag) has been theoretically predicted as a cation promoter for the A-R transition in bulk titania, although no systematic work has been reported.

Recently, the thin film properties of TiO_2_ have been thoroughly investigated, because it can be used for photocatalytic applications, as hydrophobic coating for optical glass and antireflection coating in solar cells. In addition, Ag-doped TiO_2_ films are well known for their antibacterial properties^18^. However, the influence of silver on the phase transition and other physical properties of the films is not well documented, and only a few studies have been reported on the evolution of the phases of TiO_2_ films grown by chemical methods such as sol-gel containing low Ag concentrations^19^,^20^,^21^. These works show either a mixture of TiO_2_ phases or no evidence of the rutile phase after thermal annealing at temperatures up to 700 °C^21^,^22^,^23^,^24^. In contrast, while physical deposition techniques are used such as sputtering^25^, pulsed laser^26^ and cathodic arc^27^, the influence of silver on the transition temperature from anatase to rutile of TiO_2_ films has not been fully investigated^23^,^28^.

It is evident that the tailoring of crystalline phases of TiO_2_ matrix would optimise their device performance by enhancing the catalytic properties, refractive index profile and bio-activity of the selected phase of interest^29^,^30^,^31^,^32^. It is also desirable to achieve denser rutile films at temperatures compatible with non-high temperature resistant substrates, having superior mechanical, optical and charge transport properties^33^,^34^. In a recent work, we have evaluated the effect of incorporating silver into TiO_2_ films using X-Ray Absorption Near Structure (XANES)^35^. The results had demonstrated, for the first time to the best of our knowledge, the dominant effect of silver on the phase transition from amorphous state to anatase and rutile.

The main purpose of this work is to investigate the underlying physical mechanisms of the anatase to rutile phase transformation phenomenon further and determine the effect of incorporating Ag nanopaticles into TiO_2_ thin films systematically. The phase property and surface morphology of TiO_2_ films have been studied by tailoring the addition of Ag content into TiO_2_ matrix followed by successive thermal treatments up to 1000 °C to identify the boundary of amorphous to anatase transition, and similarly the possibility of inducing anatase to rutile transition at lower annealing temperature. In addition, the wetting properties and optical spectral response of TiO_2_ thin films have been assessed to study the influence of Ag incorporation onto their photocatalytic performance and optical transmittance.

## Results

### Raman Analysis

**Figure 1:**
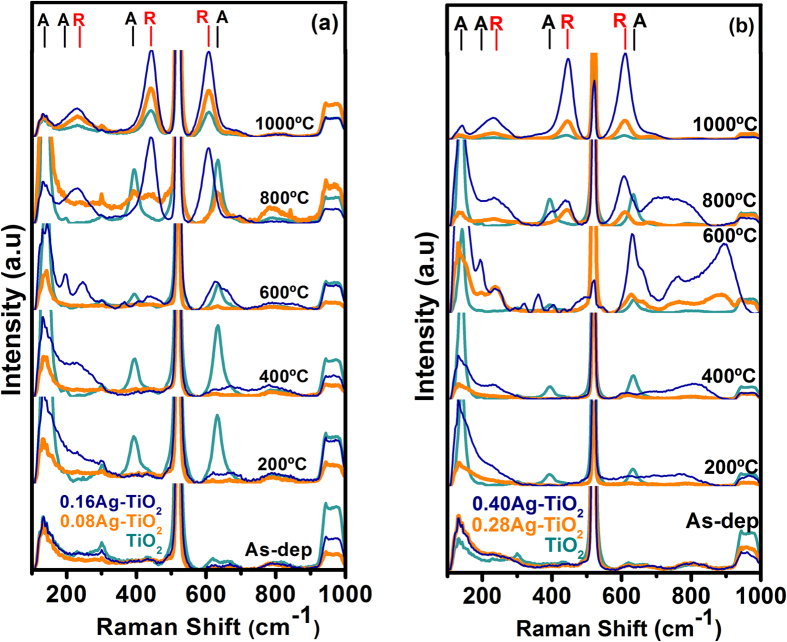
Raman spectra of the Ag-TiO_2_ films as a function of annealing temperature with silver concentration as: (**a**) 0.08–0.16Ag and (**b**) 0.28–0.40Ag. The pure TiO_2_ Raman spectrum is inserted in both graphs as reference. Anatase and Rutile bands are labelled as A and R respectively.

It is well-known that Raman spectroscopy is a powerful tool to detect the vibrational modes of chemical bonds and can identify the distinct crystalline state of materials accurately. In this work, we have employed this technique to investigate the effect of silver on the microstructural properties of TiO_2_ films after thermal annealing. The Raman spectra, as shown in Fig. 1, indicate that the as-deposited samples are non-crystalline in nature, since no characteristic bands of TiO_2_ are detected. The strong band located at 520 cm^−1^ corresponds to the LO-phonon line of the silicon substrate as well as to the associated noise as expected.

The evolution of spectra from pure TiO_2_ films following annealing, Fig. 1a, reveals three bands located at 144, 395 and 638 cm^−1^ at 200 °C that are characteristic E_g_ (Low-Frequency, LF), B_1g_, and E_g_ (High-Frequency, HF) phonon modes of anatase^36^,^37^ respectively. With an increase of the annealing temperature above 200 °C, the anatase vibrational modes became more intense until a new prominent band develops at 1000 °C, representing characteristic of vibration modes of rutile phase^38^. At this high temperature other rutile bands, 442 and 608 cm^−1^ which are associated with the E_g_ and A_1g_ phonon modes were also observed along with an additional band arising from two phonon scattering vibrations located at 235 cm^−1^.

As for the Ag-TiO_2_ nanocomposite films, the vibrational modes corresponding to the anatase start to form at 400 °C for the samples with lowest Ag content (0.08Ag-TiO_2_), whereas weaker B_1g_, and E_g_ (HF) modes of anatase tend to develop at 600 °C (Fig. 1a). Even with an increase of temperature up to 800 °C, these bands still remain weak in intensity. In addition, the E_g_ rutile vibration starts to appear at this higher temperature, which is fully developed at 1000 °C, accompanied by the evolution of the other two intense vibrational rutile modes.

For the 0.16Ag-TiO_2_ samples, Fig. 1a, the vibrational anatase modes are detected at and above 600 °C, with a new band centred at 198 cm^−1^ corresponding to the E_g_ vibration phonon mode, while the other fundamental vibrational modes of anatase are very weak. The band located at 245 cm^−1^, which is related to the double phonon scattering as stated above, indicates the anharmonicity of the emerging rutile phase^36^. Further annealing at 800 °C produces a shift of this band to 235 cm^−1^ along with other two vibrational rutile modes, the E_g_ and A_1g_ centred at 442 and 608 cm^−1^ respectively with no sign of anatase bands. At 1000 °C, only the vibrational modes associated to the rutile phase were observed.

The distinct features were observed from the 0.28Ag-TiO_2_ and 0.40Ag-TiO_2_ films unlike low Ag content samples. As shown in Fig. 1b, the broad and intense E_g_ (HF), E_g_ (198 cm^−1^) anatase modes as well as a weak rutile double phonon band (at 245 cm^−1^) were detected at 600 °C simultaneously, which were otherwise absent at lower annealing temperatures. In addition, the annealed 0.40Ag-TiO_2_ sample at 600 °C had also shown a new band at 320 cm^−1^, featuring the two-phonon scattering band of the anatase phase. Further annealing at 800 °C produces an enhancement of the vibrational rutile modes, characterized by narrow and intense bands, especially the E_g_ and A_1g_ phonon modes that became sharper when the annealing temperature reached to 1000 °C. It is noteworthy that in all of the Ag-TiO_2_ samples the broad and complex bands, located at wavenumbers higher than 650 cm^−1^, are originated from the silicon substrate.

Preliminary analysis of Raman spectra have established that the pure as-deposited TiO_2_ films grown by pulsed cathodic arc are primarily amorphous. Following thermal treatments, the anatase phase is developed at 200 °C for these films, while for Ag containing samples with the lowest atomic ratio (0.08) the anatase transition is shifted to 400 °C, and finally showing weak bands of rutile at 800 °C. In samples with Ag ratio of 0.16, anatase phase fully develops at annealing temperature of 600 °C. With increasing temperature above 600 °C, the weak rutile bands start to form that are further developed becoming more intense at 800 °C. In contrast, the spectra corresponding to samples with higher Ag ratios (0.28 and 0.40) exhibit that the samples are amorphous up to 600 °C with little sign of anatase. Above 600 °C anatase phase is predominant, however a favourable thermally induced grown condition promotes rutile phase to form and develop simultaneously unlike low Ag-content sample. At higher temperatures above 800 °C, the rutile phase seems to be the dominant one as only rutile bands are present.

### Crystallographic phase and Microstructure

**Figure 2:**
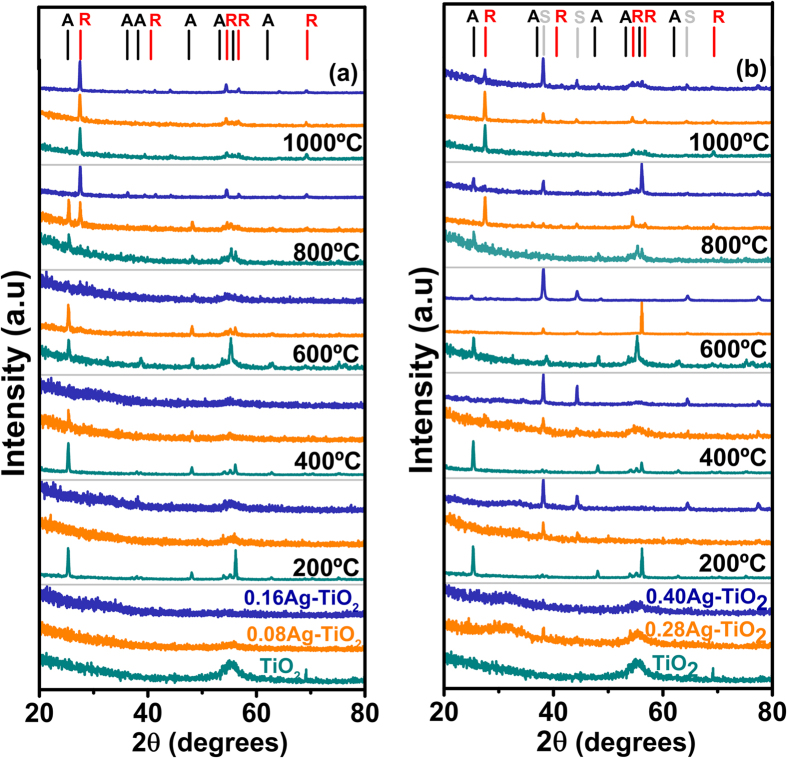
XRD patterns of pure TiO_2_ and Ag containing TiO_2_ thin films with silver concentration as: (**a**) 0.08–016Ag and (**b**) 0.28–0.40Ag annealed at different temperatures. The pure TiO_2_ XRD pattern is inserted in both graphs as reference. Anatase, Rutile and silver phases are labelled as A, R and S respectively.

Figure 2 shows grazing angle X-ray diffraction spectra of pure TiO_2_ and Ag-TiO_2_ films in as-deposited form and after annealing at temperatures up to 1000 °C. For pure TiO_2_ sample, the absence of XRD peaks confirms that the structure of the films is amorphous at room temperature (25 °C) showing only broad background, whereas above 200 °C the XRD patterns reveal four distinct peaks at 25.3, 48.1, 55.0 and 62.7° respectively, originated from anatase phase (JCPD-211272 data card, used as reference). Rutile peaks at 27.4, 54.3, 56.6 and 69.1° were only observed when the annealing temperature was increased up to 1000 °C based on rutile data card reference identification (JCPD-211276). The peak located at 56.2° is attributed to the silicon substrate.

In contrast, the XRD spectra of the Ag containing TiO_2_ films with low atomic ratio (0.08 and0.16) show that they are still amorphous at 200 °C, thereby validating Raman scan (Fig. 2a) discussed in earlier section. Only peaks related to anatase phase were observed in these samples when annealing temperature reached at 400 °C, while a mixture of rutile and anatase phases were observed at 800 °C. XRD analysis had established that the final crystal structure of the Ag-TiO_2_ films became rutile following thermal annealing at higher temperature at and above 1000 °C. This results are in agreement with previous results, which show the phase evolution of the Ag-TiO_2_ films with annealing treatments^35^.

For these low Ag-content annealed samples, no peak related to silver was observed. This can be interpreted by the fact that silver nanoparticles are finely dispersed in the film microstructure and their concentration is likely below the detection limit of the diffractometer as also reported by others^23^.

In Fig. 2b, the XRD patterns of samples with higher Ag concentrations (0.28 and 0.40 atomic ratio) reveal the formation of anatase phase only at and above 600 °C unlike the low Ag-content samples shown in Fig. 2a that produces anatase phase at 400 °C. Further thermal treatments have shown a promotion of rutile at 800 °C but with no evidence of anatase phase. At 1000 °C, only the rutile phase was observed. It was also noted from XRD analysis that high temperature annealing of the high Ag content TiO_2_ film at 1000 °C gives rise to an additional peak at 38.1°, corresponding to the (111) plane of crystalline silver (reference JCPD-40783)^39^.

**Figure 3:**
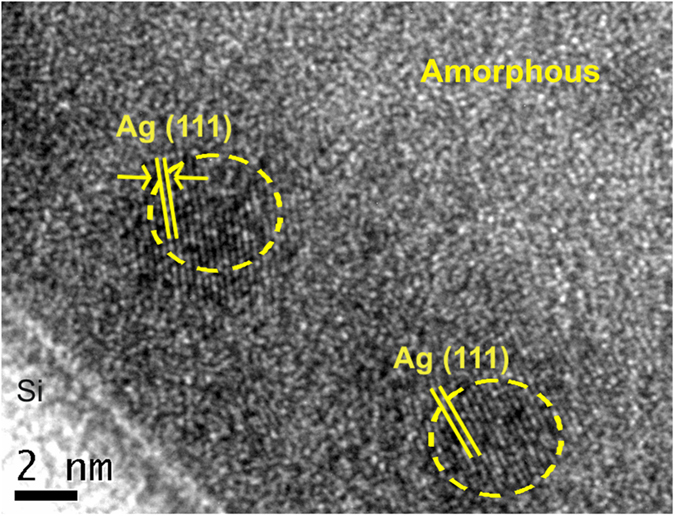
HRTEM image of silver nanoparticles in the 0.28Ag-TiO_2_ film annealed at 200 °C and of 1 hour duration, shows the (111) crystallographic planes.

The identification of the (111) silver by XRD in samples with high Ag ratio (>0.16) reveals that Ag atoms diffuse into the amorphous TiO_2_ matrix and agglomerate in forming larger clusters following heat treatment subsequently. The crystallite size of silver in the 0.28 and 0.40 Ag-TiO_2_ samples have been found to increase with the Ag atomic ratio and annealing temperature. The grain size of Ag was calculated from the width (FWHM) of the XRD peaks according to Scherrer equation as given in Table 1 40. In fact, this type of agglomeration of Ag and Au atoms as metallic nanoparticles in dielectric materials owing to heat treatment is well documented in the literature^23^. The initial nuclei are supposed to grow by Ostwald-type ripening in a broad range of sizes as the annealing temperature increases^41^. As shown in Fig. 3, HRTEM analysis exhibits the presence of Ag clusters in 0.28 Ag-TiO_2_ films following annealing at 200 °C that are of 4.5 nm size on average. In addition, TEM image also reveals the (111) crystallographic planes similar to XRD analysis, having the characteristic separation distance of 0.23 nm.

**Figure 4:**
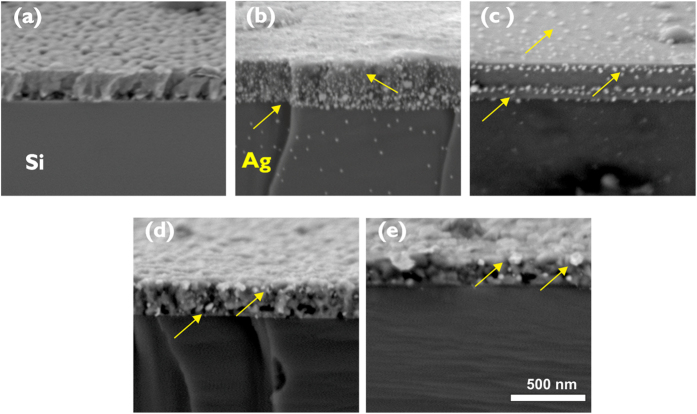
SEM images of the morphology of the 0.16Ag-TiO_2_ samples: (**a**) As-deposited and (**b**–**e**) Annealed at 200, 400, 600 and 800 °C respectively. The arrows indicate silver particles.

The formation of the Ag clusters and their agglomeration with temperature have been further identified by FESEM study of the cross sectional scan of the films. Figure 4 shows the images of silver nanoparticles embedded in Ag-TiO_2_ films annealed at different temperatures. In the as-deposited samples, the surface morphology of the film is columnar in nature with a homogeneous texture. As shown in Fig. 4b, silver nanoparticles are distributed within the coating microstructure at 200 °C, and it is agglomerated in spherical-shaped particles with a mean grain size of around 40 nm. SEM and XRD analysis had further revealed that the clustering of Ag nanoparticles increases in size from 45 to 70 nm following an increase in annealing temperature from 400 to 800 °C respectively. As observed in Fig. 4c–e, these clusters tend to diffuse and migrate towards the film surface, in agreement with the results by other authors^42^,^43^.

XRD results had confirmed the interpretation of the Raman spectra on the nature of shift of the phase transition. The transition temperature from amorphous to anatase shifts progressively up to 400 °C for low Ag content samples compared to the as-deposited TiO_2_ film, whereas such transition occurs at higher temperature at 600 °C for higher Ag concentration. It was further observed that the appearance of the rutile phase is promoted at lower temperatures 800 °C and even at 600 °C for higher Ag contents in Ag-TiO_2_ samples that would be of potential interest for thin film device applications. Furthermore, HRTEM and FESEM analysis indicate that Ag nanoparticles migrate through the TiO_2_ microstructure under the influence of thermal treatments, forming agglomerates that increase their grain size with annealing temperature.

### Silver Oxidation by XPS Analysis

**Figure 5:**
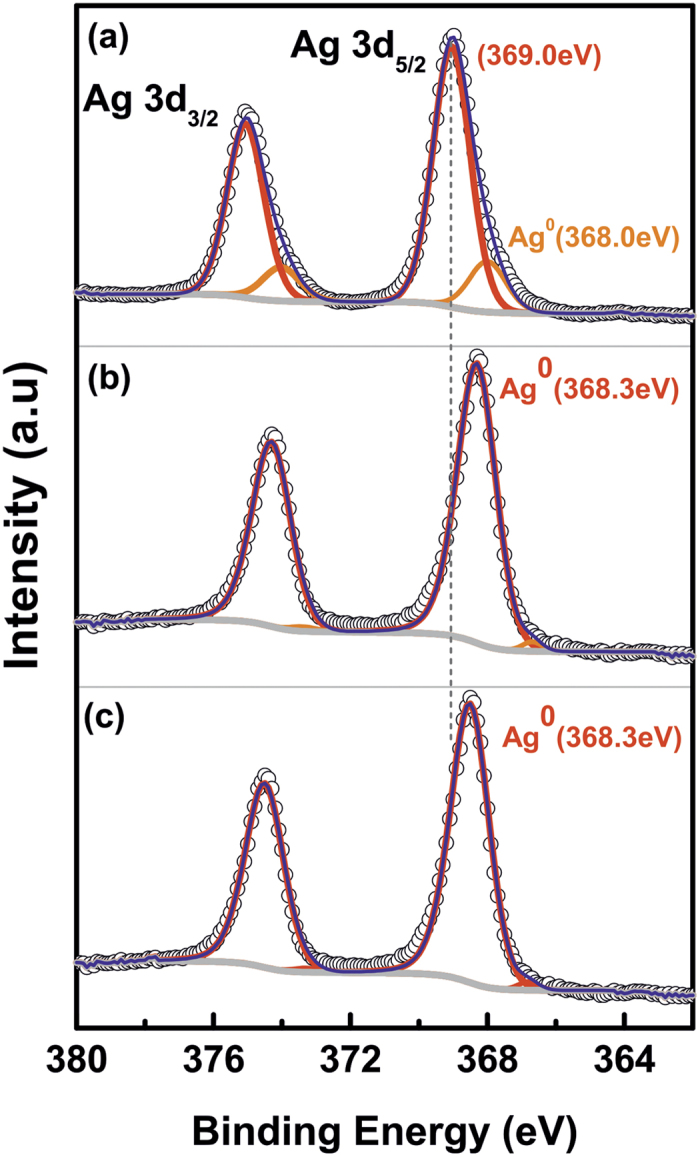
XPS Analysis of Ag-3d core level spectra of the 0.28 Ag-TiO_2_ films (**a**) As-deposited, and (**b**,**c**) Annealed at 300 °C and 400 °C respectively for 1 hour. The annealed samples have been analysed after 60 min etching.

The gradual transformation of silver to the metallic state with increasing annealing temperature had been observed by XPS analysis. Figure 5 shows the XPS spectra of the Ag 3d core level of 0.28Ag-TiO_2_ films before and after annealing at 300 and 400 °C. The Ag 3d_5/2_ and Ag 3d_3/2_ peaks have been detected giving binding energies of 369.0 and 375.0 eV respectively (Fig. 5a). After deconvolution, the spectra have shown a small shoulder peak located at 368.0 eV along with a doublet located at 374.0 eV. The high energy value of the Ag 3d peaks could be ascribed to the presence of spatial charge associated to the ion bombardment, as well as to the particle size^44^. Additionally, adventitious carbon on the film surface^45^ could also contribute to this effect, whereas the small doublet is attributed to Ag^0^ 46,47. To avoid the effect of carbon, the annealed samples were etched for 60 minutes for XPS characterisation. XPS analysis of the samples annealed at 300 °C (Fig. 5b) shows that the Ag 3d_5/2_ and Ag 3d_3/2_ core levels are located at 368.3 and 374.3 eV respectively. The position of these peaks coincides with the previous doublet, a characteristic of metallic silver^48^,^49^, which is in good agreement with the XRD results. As shown in Fig. 5c, annealing at higher temperatures allows the metallic state of silver to become stable implying that there is no significant shift of the Ag core level peaks in this case.

### Surface morphology by AFM Analysis

**Figure 6:**
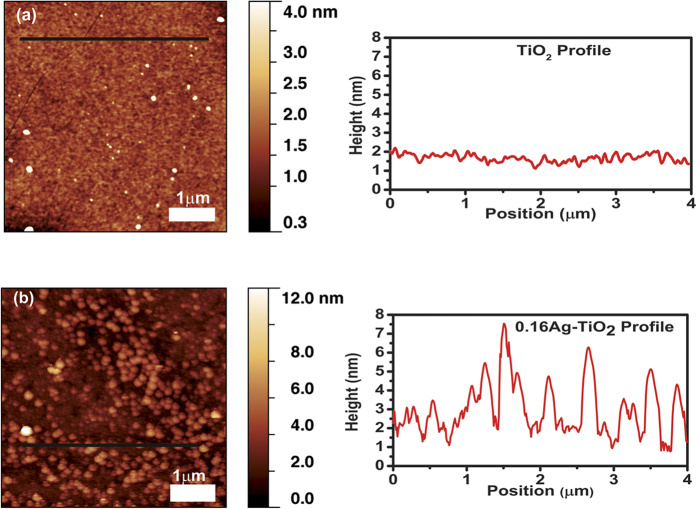
Topographic AFM images and height profile of the amorphous as-deposited films: (**a**) Pure TiO_2_ and (**b**) 0.16Ag-TiO_2_.

**Figure 7:**
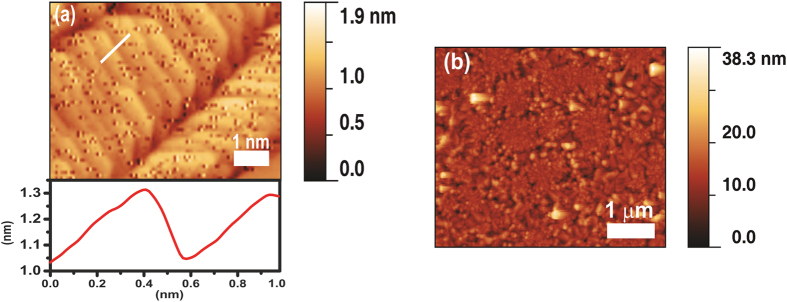
Topographic AFM images of the annealed films at 800 °C: (**a**) Pure TiO_2,_ with height profile of the atomic terraces and (**b**) 0.16Ag-TiO_2_.

Figure 6a shows the surface morphology of pure TiO_2_ and 0.16Ag-TiO_2_ films without annealing. A surface with average rms roughness of 0.2 nm (Ra = 0.2 ± 0.1 nm) was observed in the pure TiO_2_ sample. The grainy structure and the absence of atomically smooth terraces indicates that the as-deposited samples are amorphous/polycristalline in nature^50^. The 0.16Ag-TiO_2_ sample shows densely populated grain structures, giving a higher rms roughness value as Ra = 0.7 ± 0.1 nm (Fig. 6b). Following annealing at 800 °C, a change in surface morphology was clearly noticed from AFM scan of these samples. In contrast to the as-deposited pure TiO_2_, the annealed sample, Fig. 7a, shows flat terraces similar to those observed in rutile TiO_2_ (110) crystalmetals with the characteristic monoatomic step height of 0.3 ± 0.1 nm, indicating the crystallinity acquired during annelaing^51^. However, for the 0.16Ag-TiO_2_ sample, Fig. 7b, the atomic terraces are not observed which is expected due to silver diffusion and segregation onto the film surface, as confirmed by SEM analysis (Fig. 4e). In addition, a change in the average roughness value was obtained after annealing at 800 °C: the pure TiO_2_ films show Ra = 5.1 ± 0.2 nm, whereas in the 0.16Ag-TiO_2_ sample it was increased giving Ra = 7.2 ± 0.2 nm. Comparing the Ra values obtained from Ag-content samples after annealing at 800 °C, the films roughness increases slightly in good agreement with the previous results that has established this increase in Ra values related to phase transformations of the film microstructure with thermal annealing^13^,^52^.

### Wetting Property

**Figure 8:**
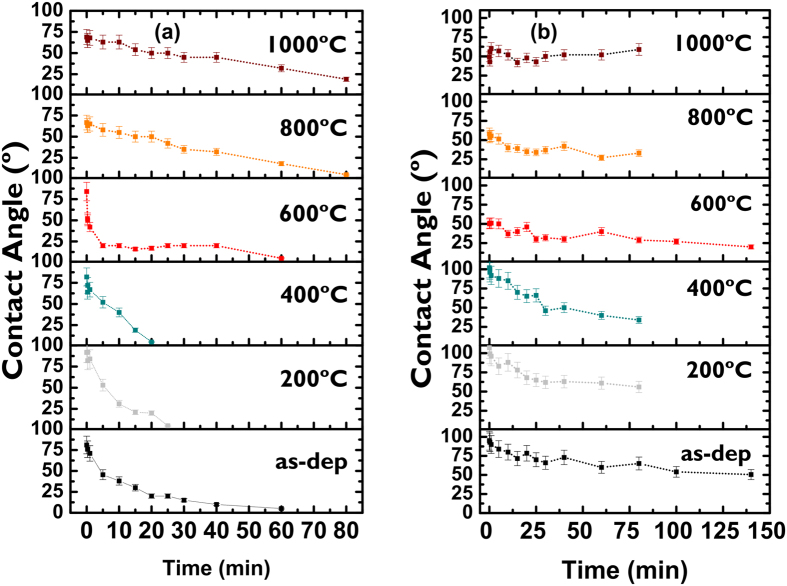
Evolution of the wetting angle on Ag-TiO_2_ films under UV illumination: (**a**) 0.08Ag-TiO_2_ and (**b**) 0.28Ag-TiO_2_.

The photocatalytic performance of the Ag-TiO_2_ films had been assessed by investigating wetting properties of the annealed samples at different temperatures. The effect of UV irradiation time on contact angle of the films is illustrated in Fig. 8a,b for two samples with different Ag contents of 0.08 and 0.28 respectively. In the as-deposited films, the value of the wetting angle before UV irradiation was measured as 81°, which indicates partially hydrophobic state of the film surface. As can be observed from Fig. 8a, the contact angle of UV-irradiated films with lower silver content (0.08) decreases monotonously in all samples irrespective of post-processing thermal treatment. This signifies the tendency of the films to become highly hydrophilic following irradiation. It is noteworthy that the contact angle decreases sharply for the samples, subjected to annealing at lower temperature range between 200–400 °C. This is, in fact, the boundary between amorphous to anatase transformation at which the film surface reaches quickly, by about 30 min, at superhydrophilic state producing contact angle ~5°, whereas the annealed samples between 600–1000 °C require longer irradiation time of about 60 min to reach the superhydrophilic state. Finally, at 1000 °C when the crystalline phase is fully transformed to rutile, the time required to achieve superhydrophilicity increases further about 80 minutes giving rise to contact angle of 19°. In contrast, the wetting angle of 0.28Ag-TiO_2_ sample shows significant difference compared to 0.08Ag-content films as shown in Fig. 8b. In higher Ag-content samples, the contact angle takes longer time up to 140 min to reach lower value in achieving hydrophilic state under similar processing conditions. In particular, the annealed samples at 600 °C and above gave a low value of contact angle, however tend to retain its hydrophilicity value,, of about 50°, substantially after prolong period of UV exposure. It is evident that the presence of Ag in TiO_2_ matrix would influence the wetting properties of the annealed film surface during crystallization process and change in microstructure induced by phase transition as reported by others^53^,^54^. The results had established that the Ag-TiO_2_ structure with high silver content, when subjected to high temperature annealing to reach full phase transformation to rutile, maintain the lower and constant value of hydrophobicity, being insensitive to UV illumination.

### Optical properties

The Ag-content samples deposited on glass substrates and annealed at 300 °C and 450 °C respectively were chosen for the spectral characterisation in the UV-visible-NIR bands. The transition temperature of the glass substrate is 557 °C that imposes limitation on choice of higher annealing temperatures. This paper is, therefore, focussed on qualitative study of the photocatalytic performance of TiO_2_ film at lower temperature range below 500 °C which prevents structural relaxation in glass to retain its mechanical property. As expected, the optical study of the TiO_2_ sample will only provide the effect of amorphous to anatase phase transformation at 300–450 °C. In fact, TiO_2_ at its anatase phase has soft crystal direction along the [001] orientation, e.g., normal to the device layer plane that allows easier band gap tuning unlike rutile phase. While the rigorous analysis of the optical properties of annealed Ag-content TiO_2_ film is beyond the scope of this paper, it will be still interesting to investigate qualitatively the degree of improvement of its photocatalytic behaviour from variation of the energy band gap (E_g_) owing to complex phase kinetics of Ag-content TiO_2_ matrix at anatase phase.

The energy band gap (E_g_) of the material was estimated from the transmission spectrum and optical absorption coefficients obtained from the well-known formulas given by equations (1,2) using graphical methods^55^.


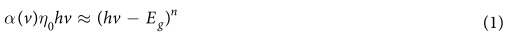



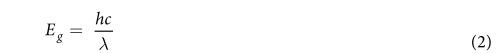


where, α is the absorption coefficient that depends on valence and conduction band configuration of the material, and varies with photon energy, *h*ν. E_g_ is the material band gap and η_0_ is the refractive index. Here, λ is wavelength, *h* is Planks constant and c is velocity of light in free space. n is a constant typically considered as 1/2 for allowed band transitions and 2 for forbidden transitions. In this study, the Ag-TiO_2_ samples annealed at 300–450 °C gave rise to anatase phase which shows an indirect band gap transition as expected. The value of constant, n is usually taken as 1/2 for allowed transition. However, for indirect and allowed transition as observed in anatase TiO_2_ film, n is considered here as 2 by taking into account of multi-phonon scattering using same approximate equation (1) as validated by others^54^.

**Figure 9:**
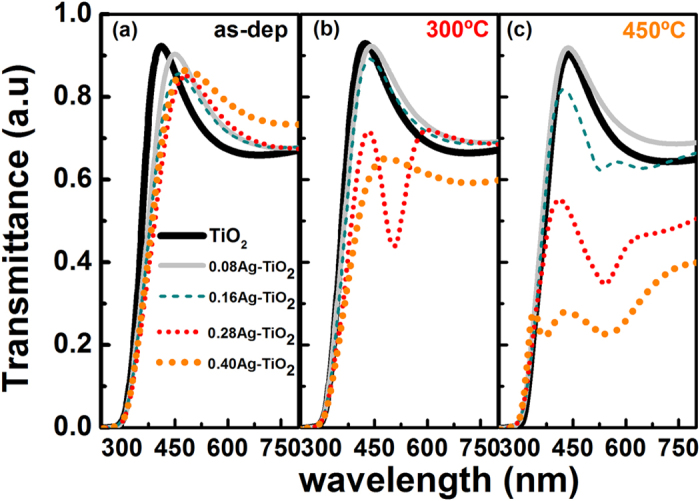
Optical transmittance spectra of TiO_2_-Ag thin film coatings deposited on glass: (**a**) As-deposited samples without annealing, (**b**) Annealed at 300 °C and (**c**) Annealed at 450 °C respectively for 1 hour.

Figure 9a–c illustrate the transmittance characteristics of the as-deposited TiO_2_ and Ag-TiO_2_ samples before and post annealing at 300 °C and 450 °C respectively. Figure 9a shows high transmittance of around 80–90% in the visible range of 350–500 nm obtained from the as-deposited TiO_2_ sample prior to annealing that starts to decrease gradually above 550 nm giving an average value of 70% in NIR band. The rapid decay of the transmittance curve for all samples towards UV band represents the intrinsic absorption band edge of the material with cut-off wavelength at λ = 390 nm. Following annealing as depicted in Fig. 9b,c, the samples exhibit similar trend with a slight shift of the transmission peak but with a reduction of transmittance values, in particular for the samples having higher Ag-content, e.g. 0.28Ag and 0.40Ag, along with a shift of the absorption band edge towards shorter wavelength. The spectral response of the higher Ag-content samples also shows an additional strong absorption band that starts to form at longer wavelength, for example the 0.28-Ag-TiO_2_ sample producing an absorption peak at 500 nm following annealing at 300 °C. This effect is prominent at annealing temperature of 450 °C for all higher Ag-content samples that give rise to a strong absorption peak with larger shift towards longer wavelength and of reduced transmission. The maximum redshift was measured from the 0.40-Ag-TiO_2_ sample with absorption peak at 547 nm. A similar nature of shift of the transmittance peak and of the absorption edge were observed for all samples depending on Ag-contents that can be attributed to thermally induced diffusion and agglomeration of Ag nanoparticles acting as scattering centres. The increased absorption and redshift towards NIR band obtained from the high Ag-content TiO_2_ structure can be explained due to the combined effects of onset of the phase transition and formation of larger Ag clusters along with surface plasmon resonance originated from Ag agglomeration and its migration towards film surface^56^.

**Figure 10:**
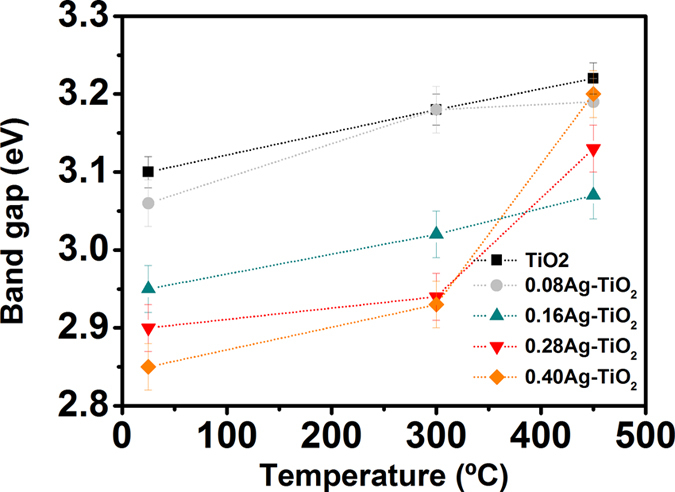
Variation of the energy bandgap (eV) versus annealing temperature for different concentration of silver content in TiO_2_ thin film structure.

Figure 10 shows the variation of bandgap (E_g_) with annealing temperature for different Ag-content TiO_2_ samples. As expected, the band gap is increased in all samples following thermal annealing due to the shift of intrinsic absorption band edge towards short wavelength. It is noteworthy that an incorporation of Ag nanoparticles in TiO_2_ matrix causes a decrease in bandgap with and without thermal treatment compared to pure TiO_2_ structure, e.g., E_g_ from 3.1 eV in TiO_2_ to 2.8 eV in 0.40Ag-TiO_2_ sample at the as-deposited condition. This can be explained by charge transfer of type-d electron of Ag to the conduction band of TiO_2_, resulting in narrowing of bandgap as reported by others^57^. In effect, Ag nanoparticles act as recombination centre for electron trapping by introducing new electronic states within bandgap of TiO_2_. Thus the preliminary results have established the possibility of improvement of photocatalytic efficiency by reducing the band gap in anatase Ag-content TiO_2_ film at lower annealing temperature.

## Discussion

The amorphous structure resulting from the deposition of compounds with highly directional bonds, such as in TiO_2_ thin films, has been attributed to the relatively high energy needed by the bonded atoms to rotate around a particular bond to satisfy crystal symmetry^58^. The energy required to re-arrange the Ti-O bonds in forming a crystalline structure, can be induced by soft thermal annealing. In this process, these bonds suffer a continuous reorganization during the transition, with the breaking and re-forming, to crystallize in anatase and eventually in the more stable rutile phase. Previous studies have shown that a relatively soft heat treatment (200–400 °C) to pure TiO_2_ films is enough to activate the transition from the amorphous to anatase phase^13^,^59^,^60^. The crystallization temperature depends on the host material, synthesis method, grain size and the deposition conditions, whereas the transition had been identified by XRD and SEM techniques^13^,^14^,^60^,^61^,^62^,^63^.

According to some authors, metal cations added to the anatase phase, with valence band lower tan +3, or larger radii than Ti^4+^ ions occupy substitutional positions in the TiO_2_ matrix. The ultimate effect is an increase in oxygen vacancies in the TiO_2_ lattice to compensate the charge neutrality, thus promoting antase-to-rutile transformation in doped samples^12^,^64^. However, such concept does not take into account of the diffusion and agglomeration processes of silver nanoparticles during the annealing treatment, as observed in this and other studies^19^,^65^,^66^.

In this work adapting cathodic arc technique, the anatase structure of films was obtained at 200 °C for pure TiO_2_, but the presence of Ag atoms increases the crystallization temperature, as detected by XRD and Raman spectroscopy. Thus, the amorphous-to-anatase transition temperature was found to be systematically delayed until it reached to 400 °C for the atomic ratio of 0.08, and to 600 °C for higher Ag concentrations. Frequently, silver is obtained in the oxide state when deposited by PVD and CVD techniques in reactive oxygen atmospheres^24^,^67^, and for this reason the increase in the transition temperature from amorphous to anatase could be ascribed to the additional energy required to dissociate Ag-O bonds initially formed in the deposited films. Additionally, the lower chemical affinity of silver towards oxygen, as compared to titanium, also favours the reduction of silver to the metallic state during annealing, as it has been revealed by XPS and XRD analysis. Moreover, FESEM image analysis indicates that Ag atoms diffuse at higher annealing temperature and eventually nucleate forming large aggregates with lower surface energy, as observed by other authors^68^.

The nucleation of silver aggregates is by itself another factor that may strongly affect the anatase transition due to their much larger size, e.g., mean grain size of several nm in annealed films at 200 °C when compared to the ionic radii of Ti and O atoms as 0.13 and 0.074 nm respectively. Therefore, it is reasonable to assume that the presence of Ag aggregates would slow down the re-arrangement of Ti-O bonds in the amorphous films to crystallize into the anatase structure. It is also found that an increase in Ag concentration tends to hinder the crystallization to higher temperatures, as indicated by XRD spectra of the 0.28 and 0.40 Ag samples, in which anatase phase forms only at 600 °C.

On the other hand, the A-R phase transformation initiates between 700–1000 °C in pure TiO_2_ powders and films^13^,^17^,^26^,^69^,^70^,^71^, while full rutile phase is usually detected at around 1000 °C. In this work, rutile phase is also clearly distinguished from anatase at 1000 °C by its characteristic vibrational bands. In case of TiO_2_ films with low Ag content (Ag below 2 at.%), some authors have observed the rutile phase at 750 °C^24^. In other studies, with lower Ag/Ti content (0.06) obtained by sol-gel, the A-R transition is found at temperatures close to 800 °C^72^. However, the samples produced in this study show an earlier onset of the transition to rutile which is initiated at lower temperature of 600 °C for the Ag atomic ratio of 0.08, with the rutile proportion in the film becoming higher as the Ag concentration increases.

The thermally induced agglomeration of silver atoms may explain the promotion of rutile phase at lower temperature, since the A-R transition implies a contraction of the c-axis, with a reduction of the unit cell volume of around 8%^12^. The agglomeration of Ag and formation of lager clusters within TiO_2_ matrix will eventually constrain the volume available for the anatase lattice, thus disrupting the anatase phase to form a more stable and denser rutile microstructure.

In addition, the presence of silver aggregates with different sizes noticeably affects the wetting properties and optical transmittance as discussed above. It is well known that the photocatalytic response in TiO_2_ is determined, among other factors, by photo-generation of electron-hole (e–h) pairs and their subsequent energy separation efficiency. According to the model proposed by Meng^68^, small size Ag clusters act as electron traps for photogenerated e–h carriers in the TiO_2_ film through a Schottky barrier effect, thus increasing the concentration of holes and, consequently, their surface energy. The ultimate effect is the absorption enhancement of –OH and O_2_^−^ species leading to hydrophilicity of the film surface as depicted in Fig. 8. On the contrary, large size Ag nanoparticles that can be found in samples with higher Ag-content and/or annealed at T > 600 °C act as electronic recombination centres. In effect, this would reduce the separation efficiency of the e-h pairs and, therefore, the hydrophilic response of the films (Fig. 8b). In addition, other morphological properties such as grain size, surface roughness, texture, and even the amorphous→anatase→rutile transition would also contribute to the wetting properties of the film surface. In particular, the surface energy of anatase has been found to be lower than rutile^12^. This may explain the lower irradiation time (20 min) needed to reach the superhydrophilic state in the samples with low Ag content, when subjected to annealing at 400 °C that promotes anatase phase in accordance with the results obtained by other authors^73^,^74^, though the films present faster response to UV irradiation. Similarly, larger size of Ag agglomerates and their diffusion towards film surface following annealing at 450 °C can be considered as the major physical mechanisms resulting in an additional absorption peak and its redshift towards NIR in high Ag content samples as observed from the transmittance spectra in Fig. 9.

**Figure 11:**
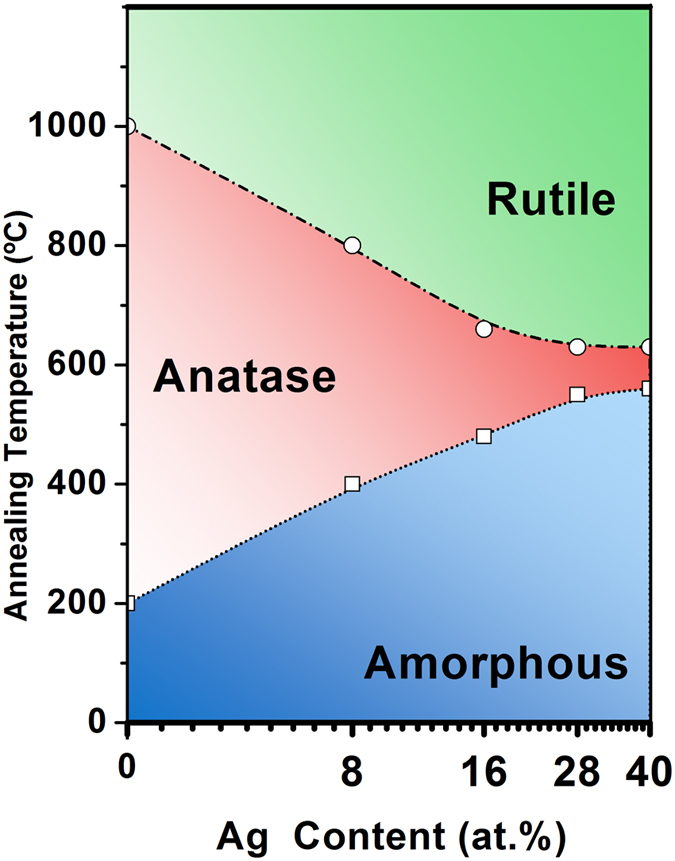
Phase diagram of the Ag-TiO_2_ films with different silver content expressed in atomic percent Ag:(Ti + Ag). The phase diagram was obtained based on both Raman and XRD data.

Finally, a qualitative description of the phase transition in Ag-TiO_2_ films is depicted schematically in Fig. 11 that demonstrates the nature of transformation of crystalline states within the Ag-TiO_2_ matrix including their interdependence on Ag atomic ratios and thermal annealing temperature, as evaluated in this interesting study. The faded zones comprise of a mixture of phases that could be found extending over a wide temperature range. It should be noted that the crystallization temperature of the different TiO_2_ phases presented in this study was taken as an estimated value since the thermal annealing was conducted for 1h each in step of 200 °C, and was increased up to 1000 °C as final step. The qualitative diagram clearly indicates an early onset of stable rutile phase at around 600 °C for higher concentration Ag-content TiO_2_ samples compared to lower Ag-content ones. This effect is envisaged to have an impact on achieving stable rutile phase in TiO_2_ films at lower processing temperature for photocatalytic and thin film device applications.

## Conclusions

The influence of silver on phase transformation of TiO_2_ films had been thoroughly investigated by depositing Ag-TiO_2_ thin film structure on silicon substrate employing reactive pulsed cathodic arc technique and followed by thermal annealing up to 1000 °C for 1 hour. The surface morphology and crystal orientation of the Ag-TiO_2_ films had been evaluated using SEM, Raman and XRD analysis with an emphasis on the wetting properties and optical transmittance. In samples with high Ag:(Ti + Ag) atomic ratio of 0.28, Raman analysis shows characteristic bands of anatase at 600 °C, co-existing with small rutile peaks which eventually develop into well defined, with no overlapping, bands of rutile at 800 °C. XRD analysis has revealed the formation of (111) plane of crystalline silver in high Ag-content sample (>0.16) indicating favourable conditions for diffusion of Ag nanoparticles into amorphous TiO_2_ structure following thermal annealing and finally, their agglomeration in forming larger clusters that has an impact on their wetting property and optical absorption. TEM analysis has identified the Ag clusters of about 4.5 nm in size estimated from 0.28 Ag-content sample at 200 °C and its (111) plane with characteristic separation of 0.23 nm. With higher annealing temperature at 800 °C, the size of Ag clusters increases up to 70 nm as observed from SEM image analysis. The experimental results have demonstrated the dominant role of Ag concentration and its grain size in tailoring thermally induced phase transition of TiO_2_ films. The amorphous to anatase transition is found to be delayed until 600 °C in low Ag-content TiO_2_ films compared to 200 °C in the pure TiO_2_ films, whereas the rutile phase is, in fact, promoted at lower temperature with onset at 600 °C for higher Ag-content TiO_2_ films.

This has established clearly the possibility of low temperature fabrication of TiO_2_ thin film structure to achieve stable rutile phase by incorporating Ag with precise control of its atomic concentration that would simultaneously (a) inhibit amorphous to anatase transition and (b) promote anatase to rutile transition at lower temperature range of 600 °C. These contrasting effects have been tentatively explained by the steric constraints imposed by diffusion and agglomeration of silver atoms in forming larger clusters following thermal annealing. In the first case, by hindering the diffusion process of the smaller titanium and oxygen ions to form anatase, whereas, in the second case, by a progressive reduction of the available space to maintain relatively high volume of the anatase unit cell, these two thermally induced processes would ultimately disrupt its anatase structure leading to formation of more stable and denser rutile phase. In addition, the study of the wetting property reveals that the presence of silver clusters in the TiO_2_ films renders their partial hydrophobic character to a superhydrophilic state, with a rapid response to the UV light, for low Ag content samples in its anatase phase, subjected to annealing at 200–400 °C. The transmittance characteristics of Ag-TiO_2_ coatings have exhibited the formation of additional absorption peak and its redshift towards near infrared in high Ag-content samples owing to large size of Ag agglomerates and surface plasmon resonance effect. The narrowing of band gap is observed in anatase Ag-content TiO_2_ films at lower annealing temperature showing promise in tailoring photocatalytic efficiency. The synergy of formation of rutile phase in TiO_2_ films at lower annealing temperature, its improved wetting properties and increased optical absorption at near infrared band by incorporation of silver nanoparticles into TiO_2_ matrix is envisaged to enhance the functionality of Ag-TiO_2_ thin films having potential for photocatalytic, antimicrobial, optical coatings and thin film IR sensor applications.

## Methods

Silver containing TiO_2_ films (Ag-TiO_2_ samples) with different silver concentration have been deposited by pulsed cathodic arc on [100] silicon substrates using pure (99.99%) titanium and silver cathodes. The setup of pulsed cathodic arc system was described elsewhere^75^,^76^. The cathodic arc technique allows the control of the composition by adjusting the pulse ratio on the Ag and Ti cathodes. The arc discharge was made in a reactive atmosphere, with an oxygen flow of 60 sccm and working pressure of 1 × 10^−2^ mbar. In order to obtain homogeneous films, the substrates were located at 12 cm from the cathodes and rotated with a frequency of 8 Hz by an MDRIVE 23 PLUS motor. The different samples, with varying Ag:Ti + Ag atomic ratio concentrations which were chosen as 0.08, 0.16, 0.28 and 0.40 respectively in trial run, were produced by changing the pulse ratio on the Ti and Ag cathodes as 38:2, 8:2, 4:2 and 2:2 respectively. The film thickness was around 100 nm in all samples, as measured with a DEKTAK 150 STYLUS profilometer. The film microstructure was analysed by scanning electron microscopy (FESEM NANOSEM 230), equipped with X-ray energy dispersion spectroscopy which was employed to estimate the Ag:(Ti + Ag) atomic ratio concentration. Following deposition, the samples were annealed in ambient atmosphere at 200, 400, 600, 800 and 1000 °C in step for 1 hour each, respectively. Raman spectra were recorded before and after thermal annealing using an ENWAVE EZraman-N spectrometer provided with a LEICA DM 300 microscope. The spectra were generated using a diode laser at 532 nm and power of 350mW. In addition, XRD analysis had been performed for all the samples, before and after annealing, by X-ray diffractometry (D8 BRUKER ADVANCED with CuKα radiation) in the 2θ range, from 20° to 80° with 0.5° incident grazing angle.

XPS measurements were performed in an ultrahigh vacuum, with a base pressure of 1 × 10^−10 ^mbar, PHOIBOS 100 ESCA/Auger spectrometer with a MgKα anode (1253.6 eV). For data analysis, the spectra were subjected to the Shirley background subtraction formalism. In order to analyze the Ag 3d core levels in the as-deposited samples, the binding energy scale was calibrated with respect to the C 1s core level peak at 285.0 eV. For the annealed samples, the spectra were collected before and after a sequence of etching treatments of about 60 minutes by Ar^+^ bombardment. The etching rate of the sample was 0.12 nm per minute.

The morphological studies of selected Ag-TiO_2_ samples before and after annealing at 800 °C were conducted using atomic force microscopy (AFM, Multimode Veeco) in the tapping mode^77^. AFM image analysis was performed with Gwyddion program applying a plane background subtraction filter. The cross section images of the thin film samples were also investigated by high resolution scanning electron microscopy (HRSEM), NANOSEM 230 FEI. Furthermore, high resolution TEM (HRTEM) images were evaluated for samples annealed at 200 °C, using a FEI TECNAI T30 microscope. The ultra-thin foils of around 25 nm were prepared by Focus Ion Beam (FIB) HELIOS 600 dual system for TEM analysis.

Finally, in order to investigate the effect of silver on the wetting properties of the films, contact angle measurements were carried out by illuminating the film surface with UV radiation (Xe lamp of 125 W) for a time period ranging from 0 up to140 min. After UV irradiation, distilled water droplets (1 μl) were produced on the samples to wet the sample surface, and the contact angle was collected with a CCD CAM 100 (KSV Instruments). The optical transmission property of TiO_2_ films was studied by depositing the thin film on borosilicate crown glass substrates and the spectral characteristics of the samples were evaluated using a SOLIDSPEC 3700 UV-visible-NIR spectrometer to calculate the transmittance and the energy band gap respectively.

## Additional Information

**How to cite this article**: Mosquera, A. A. *et al*. Effect of silver on the phase transition and wettability of titanium oxide films. *Sci. Rep*. **6**, 32171; doi: 10.1038/srep32171 (2016).

## Figures and Tables

**Table 1:** Silver (Ag) grain size (nm) in the TiO_2_ samples annealed at different temperatures.

Samples	Temperature (°C)				
	200	400	600	800	1000
0.28Ag-TiO_2_	41.4	47.7	48.1	67.5	93.4
0.40Ag-TiO_2_	45.9	55.0	63.8	42.8	66.9
